# Patterns of nonsyndromic tooth agenesis and sexual dimorphism

**DOI:** 10.1186/s12903-023-02753-1

**Published:** 2023-01-23

**Authors:** Charinya Kanchanasevee, Soranun Chantarangsu, Pisha Pittayapat, Thantrira Porntaveetus

**Affiliations:** 1 Center of Excellence in Genomics and Precision Dentistry, Department of Physiology, Faculty of Dentistry, Chulalongkorn University, Bangkok, 10330 Thailand; 2 Geriatric Dentistry and Special Patients Care Program, Faculty of Dentistry, Chulalongkorn University, Bangkok, Thailand; 3 Department of Oral Pathology, Faculty of Dentistry, Chulalongkorn University, Bangkok, Thailand; 4 Department of Radiology, Faculty of Dentistry, Chulalongkorn University, Bangkok, Thailand

**Keywords:** Anodontia, Hypodontia, Malocclusion, Microdontia, Nonsyndromic tooth agenesis, Oligodontia

## Abstract

**Background:**

Sex dimorphism has been implicated in oral health differences and the pathogenesis of oral diseases, such as tooth agenesis, periodontal disease, dental caries, and tooth loss. Tooth agenesis (TA) is one of the most common developmental anomalies in humans, and its prevalence and patterns are different across ethnic groups. The aim of this study was to investigate the phenotypes and sex-associated patterns of nonsyndromic tooth agenesis (TA) in Thai dental patients.

**Methods:**

One thousand ninety panoramic radiographs were examined. One hundred and one subjects (37 males, 64 females, 15–20 years-old) with nonsyndromic TA were evaluated. Differences in TA prevalence between groups were analyzed using the chi-square or Fisher exact test.

**Results:**

The TA prevalence, excluding third molars, was 9.3% and more frequently found in the mandible compared with the maxilla. The maxilla demonstrated a higher prevalence of first premolar agenesis than the mandible (*P* = 0.012), while the mandible had a higher prevalence of second premolar agenesis than the maxilla (*P* = 0.031). There were significantly more males missing one tooth than females, however, there were more females missing two or more teeth than males (*P* = 0.042). A missing maxillary left lateral incisor was significantly more frequent in males (*P* = 0.019), while a missing mandibular right lateral incisor was more frequent in females (*P* = 0.025). In females, the pattern of two mandibular lateral incisors agenesis was the most common and significantly present in females more than males (*P* = 0.015). In contrast, the pattern of one mandibular left lateral incisor agenesis was only observed in males and significantly found in males more than females (*P* = 0.047).

**Conclusions:**

We demonstrate sex-associated differences in nonsyndromic tooth agenesis. The prevalence of single tooth agenesis was higher in males, while that of two or more teeth agenesis was higher in females. We found different patterns of lateral incisor agenesis between males and females.

**Supplementary Information:**

The online version contains supplementary material available at 10.1186/s12903-023-02753-1.

## Background

Tooth agenesis (TA), characterized by the absence of one or more teeth, is one of the most common developmental anomalies. The TA prevalence is between 0.1 and 2.4% for the deciduous dentition and 4.4–16.2% for the permanent dentition, excluding third molars. TA has the highest prevalence in Africa (13.4%), followed by Europe (7%), Asia (6.3%), Australia (6.3%), North America (5.0%), and Latin America and the Caribbean (4.4%) [1–3]. In Asia, the prevalence is ~ 6.0–6.9% in China, 8.5–9.4% in Japan, and 7.3–11.2% in Korea [3–6]. The prevalence of hypodontia (the absence of less than six teeth) is ~ 2.3%–15.7% and that of oligodontia (the absence of more than six teeth) is ~ 0.08–1.4%, while anodontia (the complete absence of teeth) is very rare [3, 7]. The TA prevalence has been increasing in the last few decades [2, 8].

Complex interactions of genes, transcription factors, and signaling pathways regulate tooth development. TA phenotypes are genetically and phenotypically heterogeneous. TA can be isolated (nonsyndromic) or associated with other anomalies (syndromic), such as ectodermal dysplasia, Down syndrome, Axenfeld-Rieger syndrome, and Kabuki syndromes [9–11].Genetic variants causing TA are found in multiple signaling pathways, such as ectodysplasin A, wingless-type MMTV integration site family (Wnt), sonic hedgehog, bone morphogenetic protein, and fibroblast growth factor pathways [12, 13].

Disease phenotypic manifestation results from differences in genetic variations and environmental exposure between patients. Sex dimorphism has been implicated in oral health differences and the pathogenesis of oral diseases, such as TA, periodontal disease, dental caries, and tooth loss [14, 15]. Meta-analyses and many epidemiological studies have revealed that TA is significantly higher in females than males [1, 2, 16]. A study by Sato and Arai [17] demonstrated TA pattern sexual dimorphism in Japanese patients. However, the sex-associated TA patterns remain unclear. Therefore, the aim of this study was to investigate sex-associated differences in nonsyndromic TA patterns and its prevalence and characteristics in Thai dental patients, expanding the understanding between TA and gender.

## Methods

The sample size was calculated using prevalence of tooth agenesis in Thai population from the previous study [18] with an alpha = 0.05 and power = 0.08. One thousand two hundred panoramic radiographs of Thai dental patients taken between 1 January 2017 and 31 December 2017 at the Faculty of Dentistry, Chulalongkorn University, Thailand were evaluated retrospectively. The inclusion criteria were being a Thai national and 15–20 years-old on the day the radiographs were taken. The radiographs with an uncertain diagnosis, for example, the teeth might be lost due to trauma or previous extraction, were excluded. In cases with an indefinite diagnosis, previous dental history, dental casts, or any available information were examined. Subjects with congenital anomalies, records of permanent tooth extraction, trauma, or prior orthodontic treatment were excluded. Based on these criteria, 110 radiographs were excluded; resulting in 1090 evaluated panoramic radiographs that comprise 643 females and 447 males.

### Image observation

The selected radiographs were examined by an experienced dentist (C.K.) to identify the presence of tooth agenesis (excluding third molars). At least one clear panoramic radiograph of each patient was evaluated. C.K. re-evaluated 10 randomly selected panoramic radiographs (scoring 280 teeth) at a two-week minimum interval. Cohen’s kappa statistical test was performed to evaluate an intra-observer reliability. The kappa coefficient was 1.00, indicating excellent agreement. The radiographic observations were double-checked using hospital records. In case of an inconclusive finding, the radiograph was re-evaluated by another experienced dentist. If an agreement could not be obtained, the radiograph was excluded from the study. TA was defined as when tooth crown mineralization could not be identified on the panoramic radiographs. The panoramic radiographs were taken by CS8000c and CS9000c radiographic units (Carestream Health., Inc., Rochester, USA) and Veraviewepocs 3D (J. Morita, Kyoto, Japan). The standard radiographic parameters were set according to the company’s guidelines. The panoramic images were stored in the hospital picture archiving and communication system (PACS). The radiographic database was searched using Infinitt® PACS software (Infinitt Healthcare Co., Ltd., Seoul, South Korea) for panoramic radiographs that fit the inclusion criteria (subject 15–20 years-old) and radiographic date between 1 January 2017 and 31 December 2017).

### Diagnosis of TA

TA was defined as when crown mineralization could not be identified or discerned radiographically without any evidence of extraction. If a definite diagnosis of tooth agenesis could not be obtained, that radiograph was excluded. The data consisting of age, sex, location (right or left side, anterior or posterior region, maxilla or mandible), and number of missing teeth was systematically recorded. The Federation Dentaire International tooth numbering system was used to represent each tooth.

### Statistical analysis

Descriptive statistics were reported as frequencies and percentages for categorical variables. Numerical variables were presented as means with standard deviations (SD) or medians with interquartile ranges (IQR). Differences in TA prevalence between groups were analyzed using the chi-square or Fisher exact test as appropriate. To further investigate the degree of association between TA and gender, the multivariate logistic regression analysis, adjusted by age was performed. The results were reported in adjusted odds ratio (OR) with 95% confidence interval (95% CI). All analyses were performed using IBM SPSS Statistics version 22.0 (IBM). *P* < 0.05 indicated a significant difference.

## Results

Tooth agenesis, excluding third molars, was identified in 9.3% (n = 101/1090) or 8.3% of males (n = 37/447) and 9.9% of females (n = 64/643) (Additional file 1: Table S1). The average age of the patients with TA was 15.97 years (SD 0.83): 15.8 years (SD 0.91) in males and 16.02 years (SD 0.79) in females. The total number of missing teeth was 220 and the average number of missing teeth was 2.18 teeth per subject. The number of missing teeth ranged from 1 to 14 teeth (mean 2.18, SD 2.22, median 2.00, and IQR 1.00). The number of missing teeth in males ranged from 1 to 14 (mean 2.24, SD 2.89, median 1.00 and IQR 1.00), while that in females ranged from 1 to 10 (mean 2.14, SD 1.74, median 2.00 and IQR 1.00). Among the TA subjects, 43.6% had one missing tooth, 41.6% had two missing teeth, 8.9% had three to four missing teeth, and 5.9% had six or more missing teeth. We observed single tooth agenesis significantly more frequently in males compared with females while two or more teeth agenesis was significantly more frequent in females compared with males (*P* = 0.042). According to the adjusted multivariate logistic regression analysis, females were 2.36 times more likely to have two or more teeth agenesis than males (OR: 2.36; 95% CI: 1.03–5.41) (Table 1).

**Table 1:** The numbers and percentages of tooth agenesis in males and females

Number of missing teeth	Gender		Total n (% of total)	*P* value^a^	Adjusted odds ratio (95% CI)	*P* value^b^
	Males	Female				
	n (% of males)	n (% of females)				
1	21 (56.8)	23 (35.9)	44 (43.6)	***0.042***	2.36	***0.043***
2 or more	16 (43.2)	41 (64.1)	57 (56.4)		(1.03–5.41)	
Total, n (% of total)	37 (36.6)	64 (63.4)	101 (100)			

^a^Chi-square test. A significant difference (*P* < 0.05) indicates in bold italics

^b^Multivariate logistic regression analysis, adjusted by age. The male gender was the reference group

The most commonly missing teeth were the mandibular second premolars (n = 55/220), followed by the mandibular lateral incisors (n = 51/220), and the maxillary lateral incisors (n = 35/220) (Table 2). The absence of the first premolars was significantly more frequent in the maxilla than in the mandible (*P* = 0.012) while the second premolars were more frequently missing in the mandible than in the maxilla (*P* = 0.031). Overall, TA was more frequently present in the mandibular arch (61.8%, n = 136) compared with the maxillary arch (38.2%, n = 84) (Table 2). Within the same right or left side, the absence of the right first premolar was significantly more frequent in the mandibular arch than in the maxillary arch (*P* = 0.014) (Additional file 1: Table S2). Between right and left sides, differences in TA distribution for each tooth type was not significantly different (Additional file 1: Tables S3 and S4).

**Table 2:** The numbers and percentages of tooth agenesis for each tooth type in the maxillary and mandibular arches

Tooth type	Maxillary arch	Mandibular arch	Total,	Chi-square value	*P* value
	n (% of maxillary teeth)	n (% of mandibular teeth)	n (% of total)		
Central incisor	2 (2.4)	12 (8.8)	14 (6.4)	3.617	0.057^a^
Lateral incisor	35 (41.7)	51 (37.5)	86 (39.1)	0.379	0.538^a^
Canine	4 (4.7)	3 (2.2)	7 (3.2)	–	0.432^b^
First premolar	20 (23.8)	15 (11)	35 (15.9)	6.34	***0.012***^a^
Second premolar	22 (26.2)	55 (40.5)	77 (35)	4.635	***0.031***^a^
First molar	1 (1.2)	0 (0)	1 (0.4)	–	0.382^b^
Second molar	0 (0)	0 (0)	0 (0)	–	–
Total n (% of total)	84 (38.2)	136 (61.8)	220 (100)		

^a^Chi-square test

^b^Fisher exact test. A significant difference (*P* < 0.05) indicates in bold italics

**Fig. 1:**
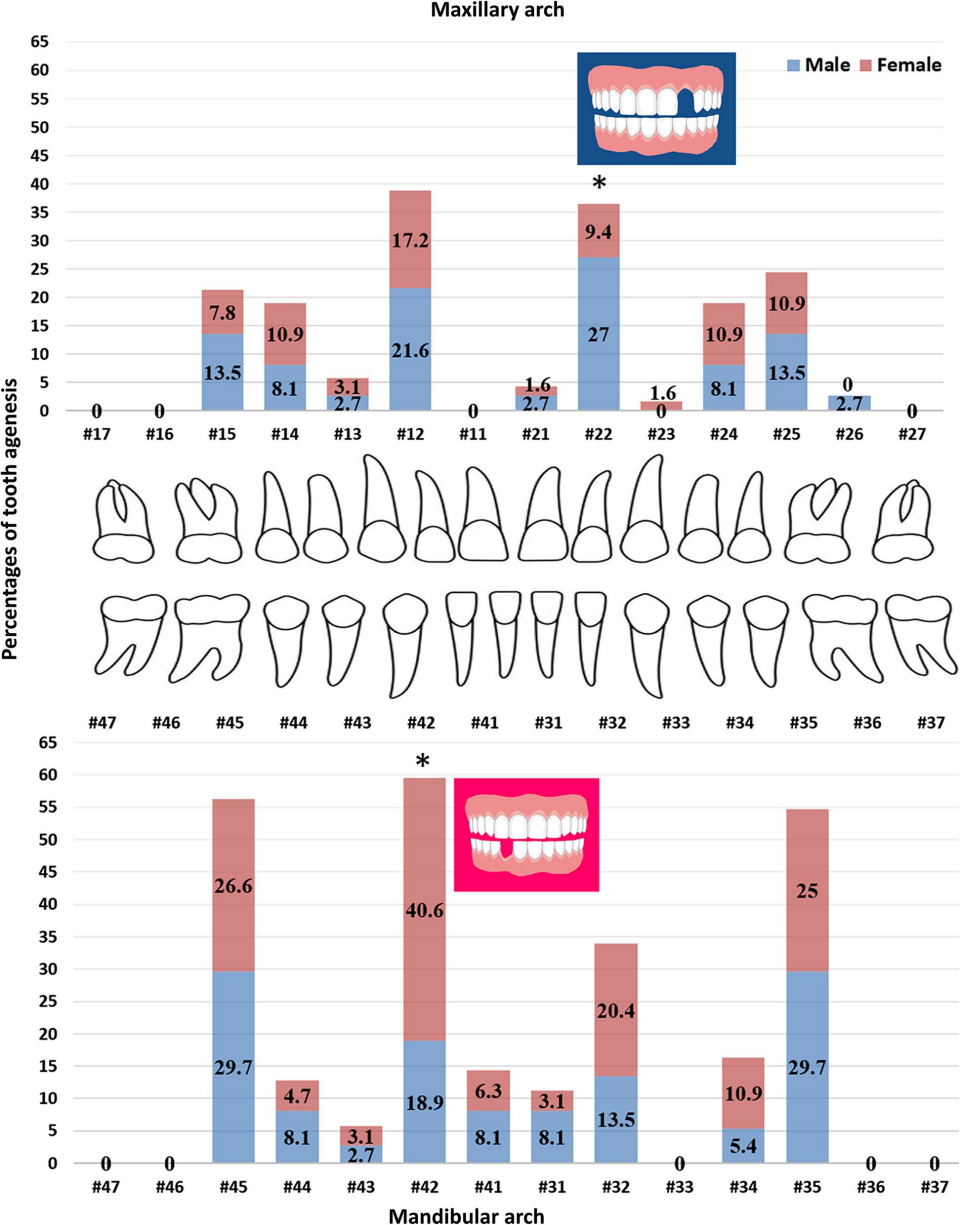
Percentage of tooth agenesis in the maxillary and mandibular arches in males and females. Percentage of the maxillary left lateral incisors agenesis was higher in males than females while the percentage of the mandibular right lateral incisors agenesis was higher in females than males. *Chi-square test indicates a significant difference (*P* < 0.05)

Considering the teeth individually, the most commonly missing teeth were the mandibular right lateral incisors (n = 33, 15%), followed by the mandibular right second premolars (n = 28, 12.7%), and the mandibular left second premolars (n = 27, 12.3%) (Table 3). Agenesis of the maxillary central incisors, the mandibular canines, the mandibular first molars, and the second molars was not observed. The absence of the maxillary left lateral incisor (#22) was significantly more frequent in males compared with females (*P* = 0.019) while agenesis of the mandibular right lateral incisor (#42) was significantly more frequent in females compared with males (*P* = 0.025). The adjusted multivariate logistic regression analysis indicated that females had agenesis of #22 less than males by 74% (OR: 0.26; 95% CI: 0.09–0.81), while agenesis of #42 in females was 2.9 times more than males (OR: 2.90; 95% CI: 1.11–7.61) (Fig. 1 and Table 3). The TA prevalence of the other teeth was not significantly different between sexes.

**Table 3:** The numbers and percentages of individual tooth agenesis in males and females

Tooth number	Gender		Total tooth number	Ranking (frequency)	Chi-square value	*P* value	Adjusted odds ratio (95% CI)	*P* value^c^
	Males	Females						
	n (% of males)	n (% of females)	n (% of total)					
#11	0 (0)	0 (0)	0 (0)		–	–	–	–
#12	8 (21.6)	11 (17.2)	19 (8.6)	4th	0.302	0.583^a^	0.74 (0.27–2.05)	0.560
#13	1 (2.7)	2 (3.1)	3 (1.4)	13th	–	1.000^b^	1.44 (0.12–17.16)	0.773
#14	3 (8.1)	7 (10.9)	10 (4.5)	8th	–	0.742^b^	1.51 (0.36–6.34)	0.575
#15	5 (13.5)	5 (7.8)	10 (4.5)	8th	–	0.491^b^	0.53 (0.14–1.97)	0.342
#16	0 (0)	0 (0)	0 (0)		–	–	–	–
#17	0 (0)	0 (0)	0 (0)		–	–	–	–
#21	1 (2.7)	1 (1.6)	2 (0.9)	14th	–	1.000^b^	0.83 (0.05–14.48)	0.900
#22	10 (27.0)	6 (9.4)	16 (7.3)	6th	5.48	***0.019***^a^	0.26 (0.09–0.81)	***0.020***
#23	0 (0)	1 (1.6)	1 (0.5)	15th	–	1.000^b^	-	-
#24	3 (8.1)	7 (10.9)	10 (4.5)	8th	–	0.742^b^	1.42 (0.34–5.88)	0.631
#25	5 (13.5)	7 (10.9)	12 (5.4)	7th	–	0.755^b^	0.81 (0.24–2.78)	0.737
#26	1 (2.7)	0 (0)	1 (0.5)	15th	–	0.366^b^	–	–
#27	0 (0)	0 (0)	0 (0)		–	–	–	–
#31	3 (8.1)	2 (3.1)	5 (2.3)	12th	–	0.353^b^	0.35 (0.05–2.21)	0.263
#32	5 (13.5)	13 (20.3)	18 (8.2)	5th	0.74	0.390^a^	1.63 (0.53–5.00)	0.398
#33	0 (0)	0 (0)	0 (0)		–	–	–	–
#34	2 (5.4)	7 (10.9)	9 (4.1)	9th	–	0.480^b^	2.28 (0.44–11.76)	0.324
#35	11 (29.7)	16 (25.0)	27 (12.3)	3rd	0.268	0.605^a^	0.79 (0.32–1.95)	0.607
#36	0 (0)	0 (0)	0 (0)		–	–	–	–
#37	0 (0)	0 (0)	0 (0)		–	–	–	–
#41	3 (8.1)	4 (6.3)	7 (3.2)	10th	–	0.705^b^	0.73 (0.15–3.49)	0.698
#42	7 (18.9)	26 (40.6)	33 (15)	1st	5.022	***0.025***^a^	2.90 (1.11–7.61)	***0.030***
#43	1 (2.7)	2 (3.1)	3 (1.4)	13th	–	1.000^b^	1.77 (0.15–21.47)	0.656
#44	3 (8.1)	3 (4.7)	6 (2.7)	11th	–	0.666^b^	0.57 (0.11–3.01)	0.509
#45	11 (29.7)	17 (26.6)	28 (12.7)	2nd	0.117	0.732^a^	0.86 (0.35–2.10)	0.734
#46	0 (0)	0 (0)	0 (0)		–	–	–	–
#47	0 (0)	0 (0)	0 (0)		–	–	–	–
Total n (% of total)	83 (37.7)	137 (62.3)	220 (100)					

^a^Chi-square test. ^b^Fisher exact test. A significant difference (*P* < 0.05) indicates in bold italics

^c^Multivariate logistic regression analysis, adjusted by age. The male gender was the reference group

**Fig. 2:**
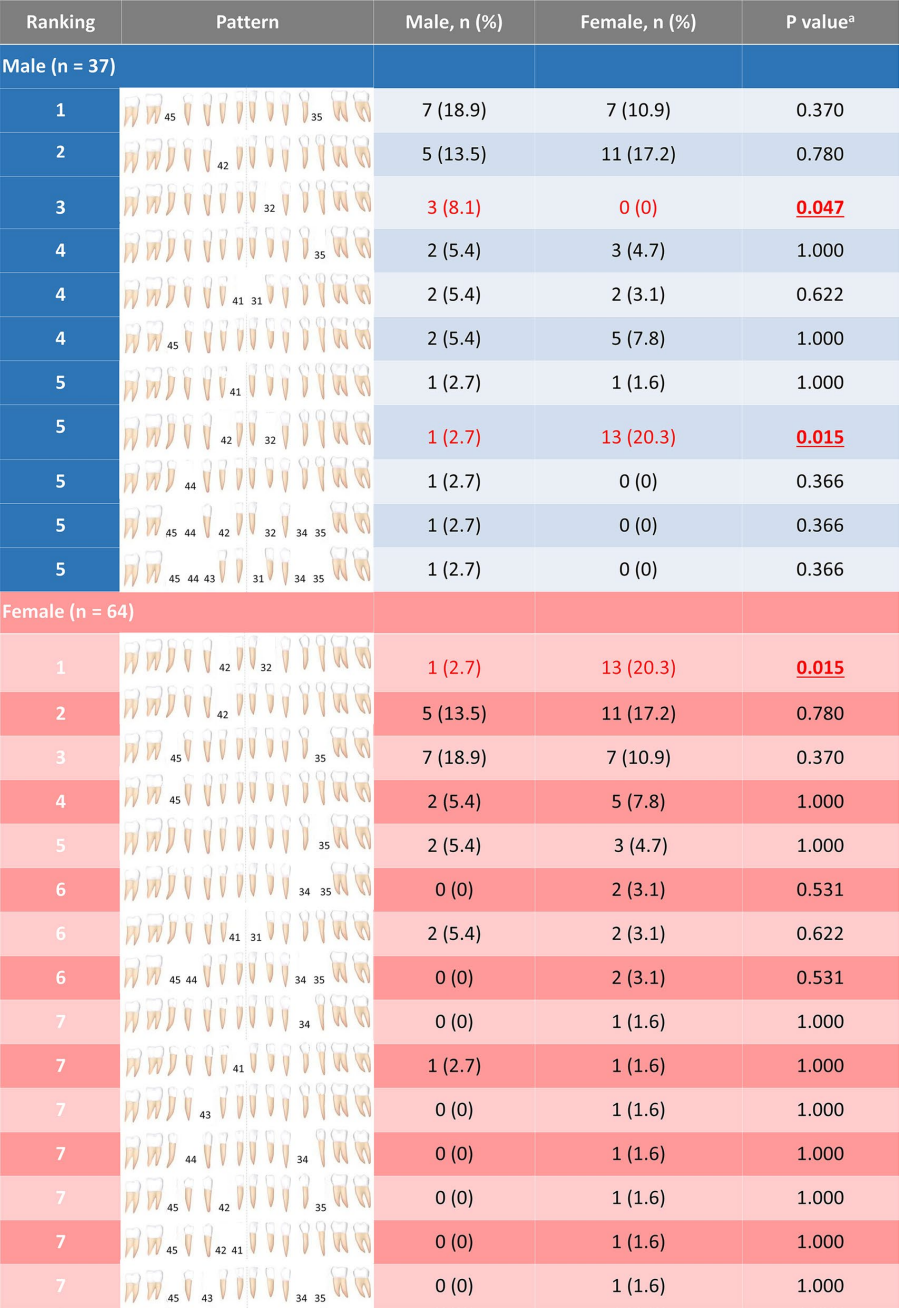
Ranking of tooth agenesis patterns of the mandibular teeth in males and females. The agenesis pattern of one mandibular left lateral incisor was significantly higher in males while the pattern of two mandibular lateral incisors missing was significantly higher in females. ^a^Chi-square test indicates a significant difference (*P* < 0.05). Missing teeth are shown tooth numbers according to the Fédération Dentaire Internationale (FDI)

**Fig. 3:**
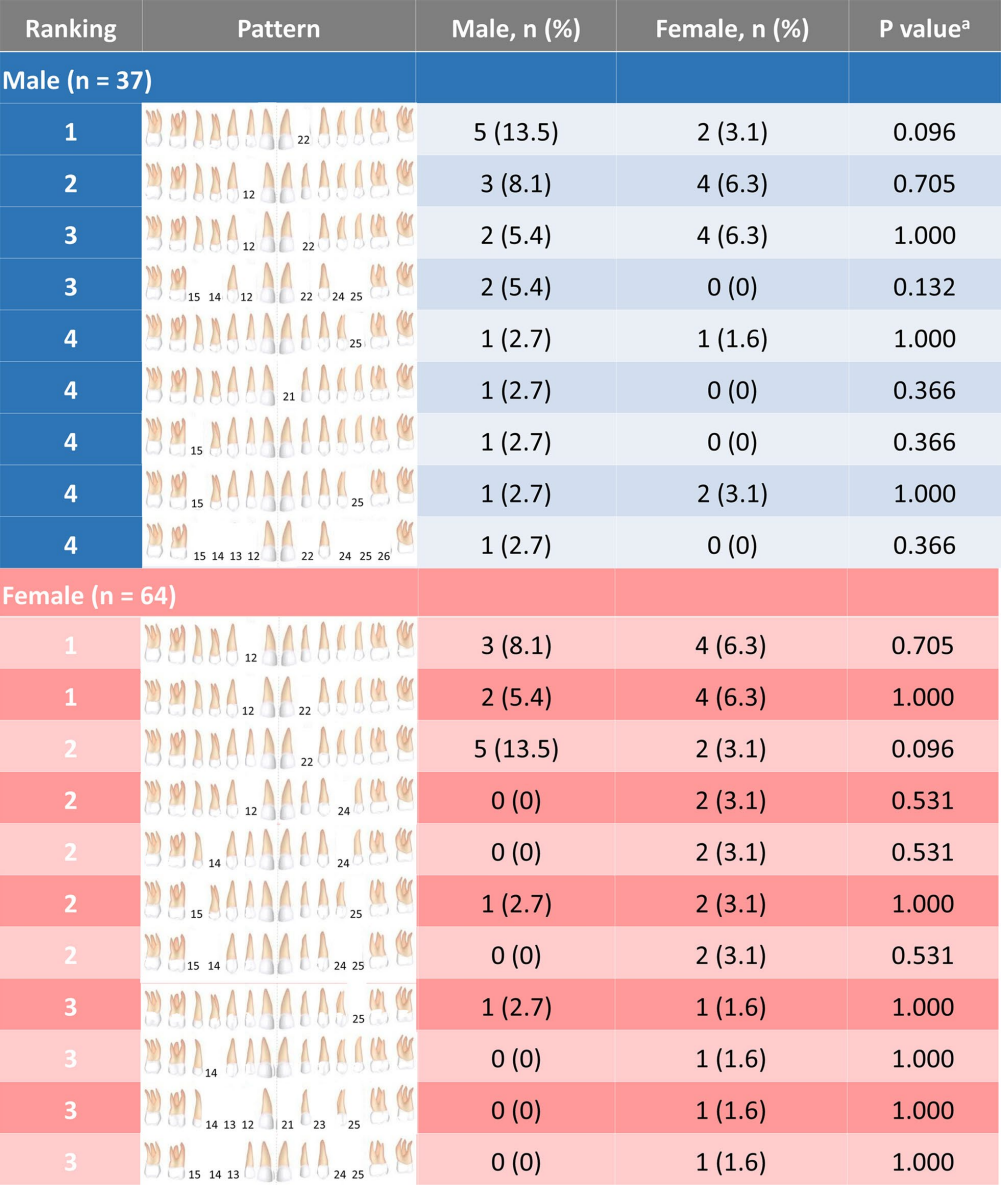
Ranking of tooth agenesis patterns of the maxillary teeth in males and females. No significant differences in the maxillary teeth agenesis pattern were observed between males and females. ^a^Chi-square test indicates a significant difference (*P* < 0.05). Missing teeth are shown FDI tooth numbers

Eighteen TA patterns were identified in males and 26 patterns in females. In the mandibular arch, 11 agenesis patterns were observed in males and 15 agenesis patterns in females. In the maxillary arch, 9 agenesis patterns were identified in males and 11 agenesis patterns in females. The maxillary and mandibular agenesis patterns were ranked and presented in Figs. 2 and 3. The three most common TA patterns in the mandibular and maxillary arches of both sexes included one lateral incisor or second premolar. In females, the pattern of two mandibular lateral incisors agenesis was the most common and significantly more frequent in females than males (*P* = 0.015). In contrast, the pattern of one mandibular left lateral incisor agenesis was only observed in males and significantly more frequent in males compared with females (*P* = 0.047). There was no significant difference in the maxillary agenesis patterns between sexes.

## Discussion

The prevalence of tooth agenesis, excluding third molars, ranges from 4.4 to 16.2%. The third molars are the most commonly absent teeth in the dentition and at least one third molar is absent in 20–30% of the population [1]. Due to its high frequency of absence, the third molars are generally excluded from TA studies. The reported TA prevalence is highly variable, which might be due to ethnic differences between populations. In Thailand, there are only 2 previous epidemiological studies concerning TA [18, 19]. Tantanapornkul [18] reported that the hypodontia prevalence was 13.7% (84 out 638 patients in the upper central area of Thailand) and the mandibular lateral incisors were the most commonly missing teeth, followed by the mandibular premolars, and the maxillary lateral incisors. Kositbowornchai et al*.* [19] reported a TA prevalence of 26.4% (150 out 570 patients in northeastern area of Thailand) and the most commonly missing teeth were the mandibular lateral incisors, followed by the maxillary lateral incisors, and the mandibular second premolars. The present study, performed in the central part of Thailand, observed a TA prevalence of 9.23%, which was lower than the previous 2 Thai studies that recruited only orthodontic patients who are more likely to have missing teeth compared with the general population. The overall prevalence of TA in Thai population is higher than the that reported in previous meta-analyses [1–3], oral health surveillance and planning in Thailand might need to be more implemented on TA to minimize its consequences on occlusion, mastication, and appearance.

In the present study, the most commonly missing teeth were the mandibular second premolars, followed by the mandibular lateral incisors, and maxillary lateral incisors. This is in accordance with meta-analysis studies and reports in Japan, Korea, and China demonstrating that the mandibular second premolars were the most frequently affected teeth, followed by the maxillary or mandibular lateral incisor, and maxillary second premolars [1, 2, 5, 6]. However, these results are different from the previous Thai studies that reported that the mandibular lateral incisors were the most commonly affected teeth [18, 19]. Although there are some discrepancies among studies, the lateral incisors and second premolars are the most commonly missing teeth after the third molars. In addition, we revealed that all TA phenotypic patterns had at least one lateral incisor or second premolar missing. In general, the agenesis was found most frequently in the third molars, followed second premolars because the second premolars and third molars are located at the distal ends of the primary dentition and the permanent (successional) dentition teeth, explained by the theory of evolution. In mammalian teeth, several theories have been proposed to explain tooth agenesis including the Butler’s field theory, Osborn’s clone model, and the homeobox code model showing that missing teeth could be a consequence of a disturbance in the dental lamina, abnormal agonistic–antagonistic signaling, or a failure of the ectomesenchyme to give rise to the adjacent tooth [20–22]. In humans, Dahlberg [23, 24] demonstrated that each tooth class had the most morphologically stable tooth (key tooth), and the other teeth are variable; for example, on the maxillary incisors, the central incisor was stable, and the lateral was variable. In general, teeth with large morphological variation are more susceptible to reduction and are more often missing. Furthermore, the development of the lateral incisors is relatively susceptible to insufficient WNT/β-catenin signaling [25]. Previous studies have demonstrated that heterozygous *WNT10A* variants are the most common variants associated nonsyndromic agenesis of second premolars and lateral incisors, and found in Thai and Asian populations more than non-Asian populations [10, 26, 27]. These can guide genetic analysis and counselling for Thai patients [12].

This study did not observe agenesis of the maxillary central incisors, the mandibular canines, the mandibular first molars, and the second molars. These findings are in accordance with previous findings showing that the maxillary central incisors, the mandibular canines, and the first molars were the teeth least affected by TA [2, 5].

When evaluating TA based on the location of the missing teeth, previous meta-analyses reported that the TA prevalence in the mandible and the maxilla was comparable [1, 2]. However, we observed that the prevalence of TA in the mandible (61.8%) was higher than the maxilla (38.2%). Moreover, we also observed that agenesis of the second premolars was significantly more frequent in the mandible compared with the maxilla while the absence of the first premolars was significantly more frequent in the maxilla compared with the mandible.

We also evaluated the effect of sex on TA and found that TA prevalence was higher in females than in males, corresponding with the previous meta-analysis study [1] and studies in Japan and Malaysia [5, 28]. We also demonstrated that the prevalence of single tooth agenesis was significantly higher in males than females while the prevalence of two or more teeth agenesis was significantly higher in females than males. Considering individual teeth, agenesis of the maxillary left lateral incisors was more frequent in males compared with females while agenesis of the mandibular right lateral incisors was more frequent in females compared with males. Sex also affected the TA phenotypic patterns, the pattern of single mandibular left lateral incisor missing was only observed in males and significantly more frequent in males than females while the pattern of two mandibular lateral incisors missing was the most common in females and significantly more frequent in females than males. These findings suggest that the development of the lateral incisors is associated with sex differences. A recent study in patients with non-syndromic oligodontia showed that females had a higher prevalence of maxillary right second premolar agenesis while males showed a higher prevalence of mandibular central incisors missing. However, they did not find sex difference in overall severity [29]. This is different from our study and previous studies that include both hypodontia and oligodontia individuals [1, 2].

Further molecular studies during tooth and dental arch development in males and females may provide greater understanding of how sex influences tooth agenesis. In addition, there seems to be a difference in the pattern of lateral incisor agenesis on the right and left sides. Studies have reported that the prevalence and pattern of right or left lateral incisor agenesis vary by race and ethnicity [30–32]. Individuals with unilateral lateral incisor agenesis had smaller teeth in the missing sides than teeth in the nonmissing sides, and smaller teeth than individuals without tooth agenesis [33, 34]. Moreover, they also had an increased prevalence of other dental anomalies such as agenesis of permanent teeth, microdontia of maxillary lateral incisors, and malpositioned teeth [35]. Unilateral agenesis of maxillary lateral incisor might provide asymmetrical support to the lip and affect smile harmony, leading to an esthetic problem. Early diagnosis of missing lateral incisors and multidisciplinary approach should be taken to ensure functional occlusion and esthetics [36].

The limitation of the study was that it was based on clinical samples with the discrepancy in the gender proportions (females were more than males). This could be because females generally seek treatment more frequently than males. The lack of a duplicate assessment of the radiographs by another examiner is a limitation to the current findings. However, the radiographic findings were confirmed with the hospital records. Moreover, the intra-examiner reliability with kappa score of 1 indicates perfect agreement. Moreover, TA was evaluated by using panoramic radiographs of the subjects aged 15–20 years who were likely to attend the University hospital for orthodontic treatment. Our findings should be confirmed in the general population to better represent TA characteristics of Thai people in a further study.

## Conclusions

The study demonstrates sexual differences in tooth agenesis patterns. The prevalence of single tooth agenesis was higher in males while that of two or more teeth agenesis was higher in females. In addition, there was a significant difference in agenesis of the lateral incisors between males and females. The identification of various tooth agenesis patterns and gender related tendencies suggest that the categories might be utilized in future epidemiological studies. Further molecular genetic studies may provide better understating of how sex influences tooth agenesis.

## Supplementary Information

**Additional file 1.** **Supplementary Data. Supplementary Table 1.** The prevalence of tooth agenesis. **Supplementary Table 2.** The number and percentage of tooth agenesis for each tooth type in the same side (right or left) between the maxillary and mandibular arches. **Supplementary Table 3.** The number and percentage of tooth agenesis for each tooth type in the right and left sides. **Supplementary Table 4.** The number and percentage of tooth agenesis for each tooth type in the same dental arch (maxillary or mandibular) between the right and left sides.

## Data Availability

All data generated or analysed during this study are included in this published article and its Additional file 1.

## Abbreviations

IQR: Interquartile ranges
PACs: Picture archiving and communication system
SD: Standard deviation
TA: Tooth agenesis
WNT: Wingless
