# Supplementary material for: Patterns of nonsyndromic tooth agenesis and sexual dimorphism
Source: BMC Oral Health. 2023 Jan 23;23:37. doi: 10.1186/s12903-023-02753-1 (PMC9869554; doi:10.1186/s12903-023-02753-1)
Supplement: Supplementary file 1 — Additional file 1. Supplementary Data. Supplementary Table 1. The prevalence of tooth agenesis. Supplementary Table 2. The number and percentage of tooth agenesis for each tooth type in the same side (right or left) between the maxillary and mandibular arches. Supplementary Table 3. The number and percentage of tooth agenesis for each tooth type in the right and left sides. Supplementary Table 4. The number and percentage of tooth agenesis for each tooth type in the same dental arch (maxillary or mandibular) between the right and left sides. [file 12903_2023_2753_MOESM1_ESM.pdf]

Supplementary Data

Patterns of nonsyndromic tooth agenesis and sexual dimorphism

Charinya Kanchanasevee[1,2], Soranun Chantarangsu[3], Pisha Pittayapat[4], Thantrira Porntaveetus[1,*]

[1]Center of Excellence in Genomics and Precision Dentistry, Department of Physiology, Faculty of Dentistry, Chulalongkorn University, Bangkok, Thailand

[2]Geriatric Dentistry and Special Patients Care Program, Faculty of Dentistry, Chulalongkorn University, Bangkok, 10330, Thailand

[3]Department of Oral Pathology, Faculty of Dentistry, Chulalongkorn University, Bangkok, Thailand

[4]Department of Radiology, Faculty of Dentistry, Chulalongkorn University, Bangkok, Thailand

*Correspondence to:

Thantrira Porntaveetus, DDS, Grad Dip, MSc, PhD. ORCID number: 0000000301459801

Genomics and Precision Dentistry Research Unit, Department of Physiology, Faculty of Dentistry, Chulalongkorn University, Bangkok 10330, Thailand

Tel: 662-218-8695; Fax: 662-218-8691; E-mail: thantrira.p@chula.ac.th

Short running title: Tooth agenesis and sexual dimorphism

ORCID: CK: 0000-0001-7107-0709, SC: 0000-0002-3400-5585, PP: 0000-0001-9607-5450, TP: 0000-0003-0145-9801

Supplementary Table 1. The prevalence of tooth agenesis.

Gender	Number of patients		Prevalence (%)
	Examined (n)	Affected (n)	
Male	447	37	8.3
Female	643	64	9.9
Total	1090	101	9.3

Supplementary Table 2. The number and percentage of tooth agenesis for each tooth type in the same side (right or left) between the maxillary and mandibular arches.

Maxillary arch		Mandibular arch			Maxillary arch		Mandibular arch		
Tooth number	Number, n (% of maxillary right teeth)	Tooth number	Number, n (% of mandibular right teeth)	*P* value	Tooth number	Number, n (% of maxillary left teeth)	Tooth number	Number, n (% of mandibular left teeth)	*P* value
#11	0 (0)	#41	7 (9.1)	0.051[b]	#21	2 (4.7)	#31	5 (8.5)	0.696[b]
#12	19 (45.2)	#42	33 (42.8)	0.802[a]	#22	16 (38.1)	#32	18 (30.5)	0.426[a]
#13	3 (7.2)	#43	3 (3.9)	0.664[b]	#23	1 (2.4)	#33	0 (0)	0.416[b]
#14	10 (23.8)	#44	6 (7.8)	**<u>0.014</u>**[a]	#24	10 (23.8)	#34	9 (15.2)	0.278[a]
#15	10 (23.8)	#45	28 (36.4)	0.160[a]	#25	12 (28.6)	#35	27 (45.8)	0.080[a]
#16	0 (0)	#46	0 (0)	-	#26	1 (2.4)	#36	0 (0)	0.416[b]
#17	0 (0)	#47	0 (0)	-	#27	0 (0)	#37	0 (0)	-

[a]Chi-square test. [b]Fisher exact test. A significant difference ($P < 0.05$) indicates in bold and underlined.

Supplementary Table 3. The number and percentage of tooth agenesis for each tooth type in the right and left sides.

Tooth type	Right side, n (% of right teeth)	Left side, n (% of left teeth)	Total, n (% of total)	Chi-square value	*P* value
Central incisor	7 (5.9)	7 (6.9)	14 (6.4)	0.101	0.751[a]
Lateral incisor	52 (43.7)	34 (33.7)	86 (39.1)	2.310	0.129[a]
Canine	6 (5)	1 (1)	7 (3.2)	-	0.128[b]
First premolar	16 (13.5)	19 (18.8)	35 (15.9)	1.176	0.278[a]
Second premolar	38 (31.9)	39 (38.6)	77 (35)	1.072	0.301[a]
First molar	0 (0)	1 (1)	1 (0.4)	-	0.459[b]
Second molar	0 (0)	0 (0)	0 (0)	-	-
Total, n (% of total)	119 (54.1)	101 (45.9)	220 (100)		

[a]Chi-square test. [b]Fisher exact test

Supplementary Table 4. The number and percentage of tooth agenesis for each tooth type in the same dental arch (maxillary or mandibular) between the right and left sides.

Maxillary arch					Mandibular arch				
Right side		Left Side			Right Side		Left Side		
Tooth number	Number, n (% of maxillary right teeth)	Tooth number	Number, n (% of maxillary left teeth)	*P* value	Tooth number	Number, n (% of mandibular right teeth)	Tooth number	Number, n (% of mandibular left teeth)	*P* value
#11	0 (0)	#21	2 (4.7)	0.494[b]	#41	7 (9.1)	#31	5 (8.5)	0.900[a]
#12	19 (45.2)	#22	16 (38.1)	0.507[a]	#42	33 (42.8)	#32	18 (30.5)	0.140[a]
#13	3 (7.2)	#23	1 (2.4)	0.616[b]	#43	3 (3.9)	#33	0 (0)	0.258[b]
#14	10 (23.8)	#24	10 (23.8)	1.000[a]	#44	6 (7.8)	#34	9 (15.2)	0.169[a]
#15	10 (23.8)	#25	12 (28.6)	0.620[a]	#45	28 (36.4)	#35	27 (45.8)	0.268[a]
#16	0 (0)	#26	1 (2.4)	1.000[b]	#46	0 (0)	#36	0 (0)	-
#17	0 (0)	#27	0 (0)	-	#47	0 (0)	#37	0 (0)	-

[a]Chi-square test. [b]Fisher exact test.
